# Characterizing microbiota-independent effects of oligosaccharides on intestinal epithelial cells: insight into the role of structure and size: Structure–activity relationships of non-digestible oligosaccharides

**DOI:** 10.1007/s00394-016-1234-9

**Published:** 2016-06-13

**Authors:** Peyman Akbari, Johanna Fink-Gremmels, Rianne H. A. M. Willems, Elisabetta Difilippo, Henk A. Schols, Margriet H. C. Schoterman, Johan Garssen, Saskia Braber

**Affiliations:** 1 Division of Veterinary Pharmacology, Pharmacotherapy and Toxicology, Institute for Risk Assessment Sciences, Utrecht University, Yalelaan 104, 3584 CM Utrecht, The Netherlands; 2 Division of Pharmacology, Utrecht Institute for Pharmaceutical Sciences Faculty of Science, Utrecht University, 3584 CG Utrecht, The Netherlands; 3 Laboratory of Food Chemistry, Wageningen University, 6708 WG Wageningen, The Netherlands; 4 FrieslandCampina, 3818 LE Amersfoort, The Netherlands; 5 Nutricia Research, 3584 CT Utrecht, The Netherlands

**Keywords:** Caco-2 cells, CXCL8, Degree of polymerization, Intestinal permeability, Non-digestible oligosaccharides, Tight junctions

## Abstract

**Purpose:**

The direct effects of galacto-oligosaccharides (GOS), including Vivinal^®^ GOS syrup (VGOS) and purified Vivinal^®^ GOS (PGOS), on the epithelial integrity and corresponding interleukin-8 (IL-8/CXCL8) release were examined in a Caco-2 cell model for intestinal barrier dysfunction. To investigate structure–activity relationships, the effects of individual DP fractions of VGOS were evaluated. Moreover, the obtained results with GOS were compared with Caco-2 monolayers incubated with fructo-oligosaccharides (FOS) and inulin.

**Methods:**

Caco-2 monolayers were pretreated (24 h) with or without specific oligosaccharides or DP fractions of VGOS (DP2 to DP6) before being exposed for 12 or 24 h to the fungal toxin deoxynivalenol (DON). Transepithelial electrical resistance and lucifer yellow permeability were measured to investigate barrier integrity. A calcium switch assay was used to study the reassembly of tight junction proteins. Release of CXCL8, a typical marker for inflammation, was quantified by ELISA.

**Results:**

In comparison with PGOS, FOS and inulin, VGOS showed the most pronounced protective effect on the DON-induced impairment of the monolayer integrity, acceleration of the tight junction reassembly and the subsequent CXCL8 release. DP2 and DP3 in concentrations occurring in VGOS prevented the DON-induced epithelial barrier disruption, which could be related to their high prevalence in VGOS. However, no effects of the separate DP GOS fractions were observed on CXCL8 release.

**Conclusions:**

This comparative study demonstrates the direct, microbiota-independent effects of oligosaccharides on the intestinal barrier function and shows the differences between individual galacto- and fructo-oligosaccharides. This microbiota-independent effect of oligosaccharides depends on the oligosaccharide structure, DP length and concentration.

**Electronic supplementary material:**

The online version of this article (doi:10.1007/s00394-016-1234-9) contains supplementary material, which is available to authorized users.

## Introduction

Human milk oligosaccharides (HMOs) play an essential role in the postnatal growth and development of the intestinal and immune system [1]. As the sufficient supply of the neonate with HMOs cannot always be guaranteed, various attempts were made to design alternative prebiotic oligosaccharides that mimic the gut health-promoting effects of HMOs. At present, prebiotic oligosaccharides, for example the mixture of 90 % GOS/10 % lcFOS, aiming to mimic molecular size distribution of neutral HMOs, are widely used in infant formulas [2, 3]. Although these neutral oligosaccharides are structurally different from HMOs, they have prebiotic activities, and clinical investigations have confirmed that infants given such a formula containing GOS or GOS/lcFOS achieve an intestinal microbiota composition comparable to that of breastfed infants [4, 5]. Besides the effects of GOS and the mixture of GOS/lcFOS on the gut microbiota [6, 7], direct interactions of these oligosaccharides with intestinal epithelial cells have recently been described by our group and others [8–11]. It has been shown that these oligosaccharides improve and protect the intestinal barrier integrity and modulate the immune responses of epithelial cells.

GOS and FOS differ in origin and structure, and it has been suggested previously that the biological function of oligosaccharides is influenced by their structures, molecular weight and type of glycosidic linkages [12, 13]. GOS are oligosaccharides based on the milk sugar lactose, and the oligomers [degree of polymerization (DP)2–8] are produced by glycosylation of lactose using the enzyme β-galactosidase [14]. Inulin, also called long-chain FOS (lcFOS), corresponds to unprocessed chicory inulin, mainly composed of fructans (DP2-60) ending with a terminal glucose monomer, whereas FOS are composed of partially hydrolyzed inulin (DP2-8), and more molecules end with a fructose rather than with a glucose monomer [15, 16]. In addition to origin and structure, the specific DP composition is believed to influence the prebiotic activity and the degree of fermentation of oligosaccharides, since distinct sensitivity of human gut bacteria to selected DP of oligosaccharides has been observed [13, 17, 18]. However, the direct interactions of individual DP fractions with intestinal epithelial cells have not yet been investigated.

In the current study, we aimed to compare direct, microbiota-independent effects of different galacto-oligosaccharides, on dysregulated intestinal epithelial barrier function and the related inflammatory response. Monolayers of the human intestinal epithelial cell line, Caco-2, served as a model system for intestinal barrier function, while the fungal toxin deoxynivalenol (DON) was used as a model compound to impair the intestinal integrity as previously described by Akbari et al. [19]. Considering the protective effects of GOS in this Caco-2 cell model, we compared these results to the effects of FOS and inulin and further evaluated the effect of individual DP fractions of GOS (ranging from DP2 to DP6).

## Materials and methods

### Deoxynivalenol (DON)

Purified DON (D0156; Sigma-Aldrich, St Luis, MO, USA) was dissolved in pure ethanol (99.9 %, JT Baker, Deventer, The Netherlands) and stored at −20 °C. The mycotoxin DON was selected as model compound to impair intestinal barrier integrity in the cell culture experiments. DON was diluted to a concentration of 4.2 μM in complete cell culture medium and added to the apical side as well as to the basolateral side of the transwell inserts for either 12 or 24 h. This DON concentration was selected on the basis of our previous results and did not affect the viability of the Caco-2 cells [19].

### Oligosaccharides

**Fig. 1:**
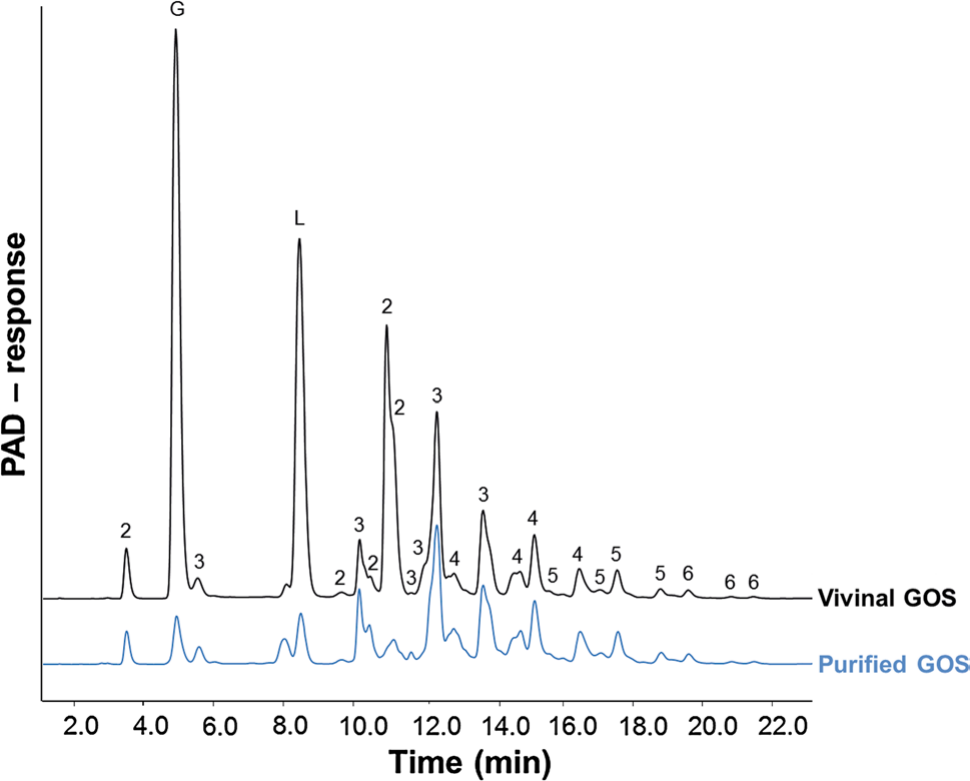
VGOS and PGOS characteristics. HPAEC-PAD elution patterns of Vivinal^®^ GOS syrup and purified GOS. Numbers *2*–*6* correspond to galacto-oligosaccharides having a degree of polymerization from 2 to 6. *G* and *L* represent galactose/glucose and lactose, respectively

**Fig. 2:**
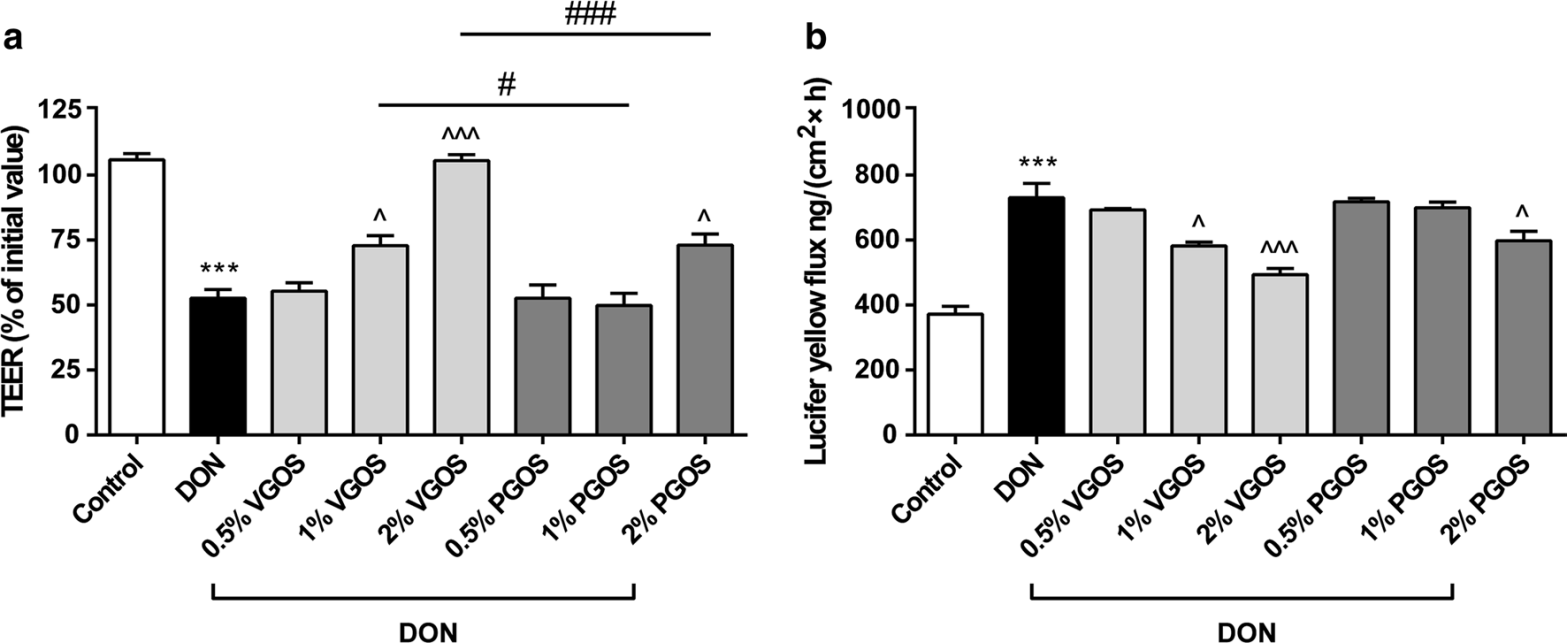
Different effects of VGOS and PGOS on the DON-induced impairment of the Caco-2 cell monolayer integrity. Caco-2 cells were pretreated apically and basolaterally with increasing concentrations (0.5, 1 and 2 %) of VGOS or PGOS (24 h) prior to the addition of DON (4.2 μM) (apical and basolateral compartments) for another 24 h. Subsequently, the TEER (**a**) and the translocation of lucifer yellow from the apical to the basolateral compartment (**b**) were measured. Results are expressed as a percentage of initial value (TEER) or the amount of tracer transported [ng/(cm^2^ × h)] as mean ± SEM of three independent experiments, each performed in triplicate (***P < 0.001: significantly different from the unstimulated cells; ^*P* < 0.05, ^^^*P* < 0.001: significantly different from the DON-stimulated cells; ^#^ *P* < 0.05, ^###^ *P* < 0.001: significantly different from each other)

**Fig. 3:**
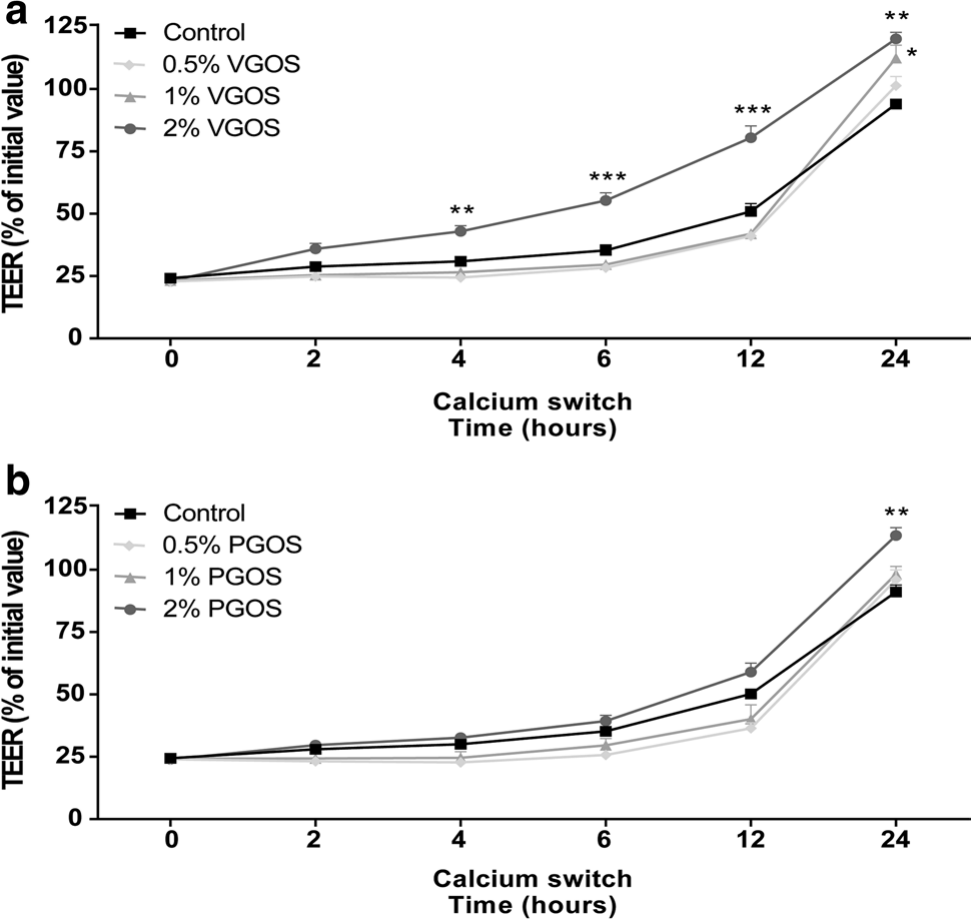
VGOS time-dependently accelerated tight junction reassembly after calcium deprivation in Caco-2 cells. Caco-2 cells were pretreated apically and basolaterally with increasing concentrations (0.5, 1 and 2 %) of VGOS (**a**) or PGOS (**b**) (24 h) prior to transient calcium deprivation with HBSS-EGTA to disrupt tight junction proteins. TEER was measured at the indicated time points (0, 2, 4, 6, 12 and 24 h) during recovery in complete, calcium-containing DMEM with either VGOS (**a**) or PGOS (**b**). Results are expressed as a percentage of initial value as mean ± SEM of three independent experiments, each performed in triplicate (*P < 0.05, **P < 0.01, ***P < 0.001: significantly different from the untreated cells)

**Fig. 4:**
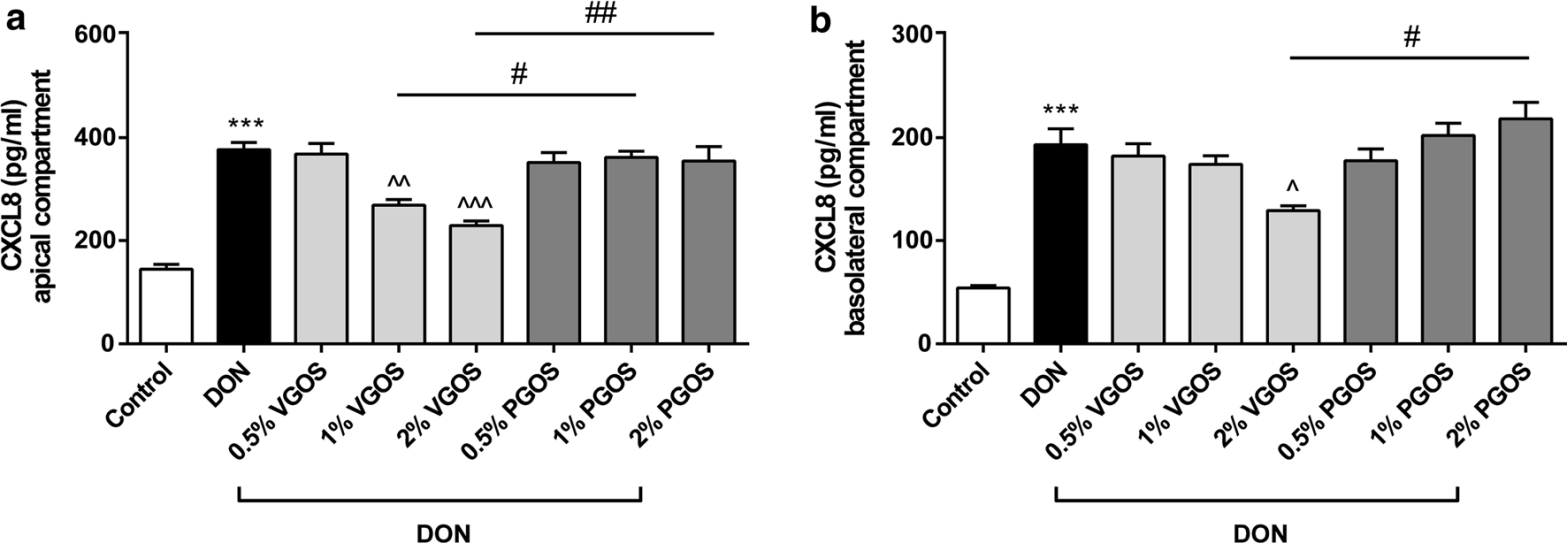
VGOS was able to suppress the DON-induced increase in CXCL8 release by Caco-2 cells. Caco-2 cells were pretreated apically and basolaterally with increasing concentrations (0.5, 1 and 2 %) of VGOS or PGOS (24 h) prior to the addition of DON (4.2 μM) (apical and basolateral compartments) for 24 h. CXCL8 secretion into medium of apical (**a**) and basolateral (**b**) compartments was measured by ELISA. Results are expressed as pg/ml as mean ± SEM of three independent experiments, each performed in triplicate (***P < 0.001; significantly different from the unstimulated cells. ^*P* < 0.05, ^^*P* < 0.01, ^^^*P* < 0.001: significantly different from the DON-stimulated cells; ^#^ *P* < 0.05, ^##^ *P* < 0.01: significantly different from each other)

**Fig. 5:**
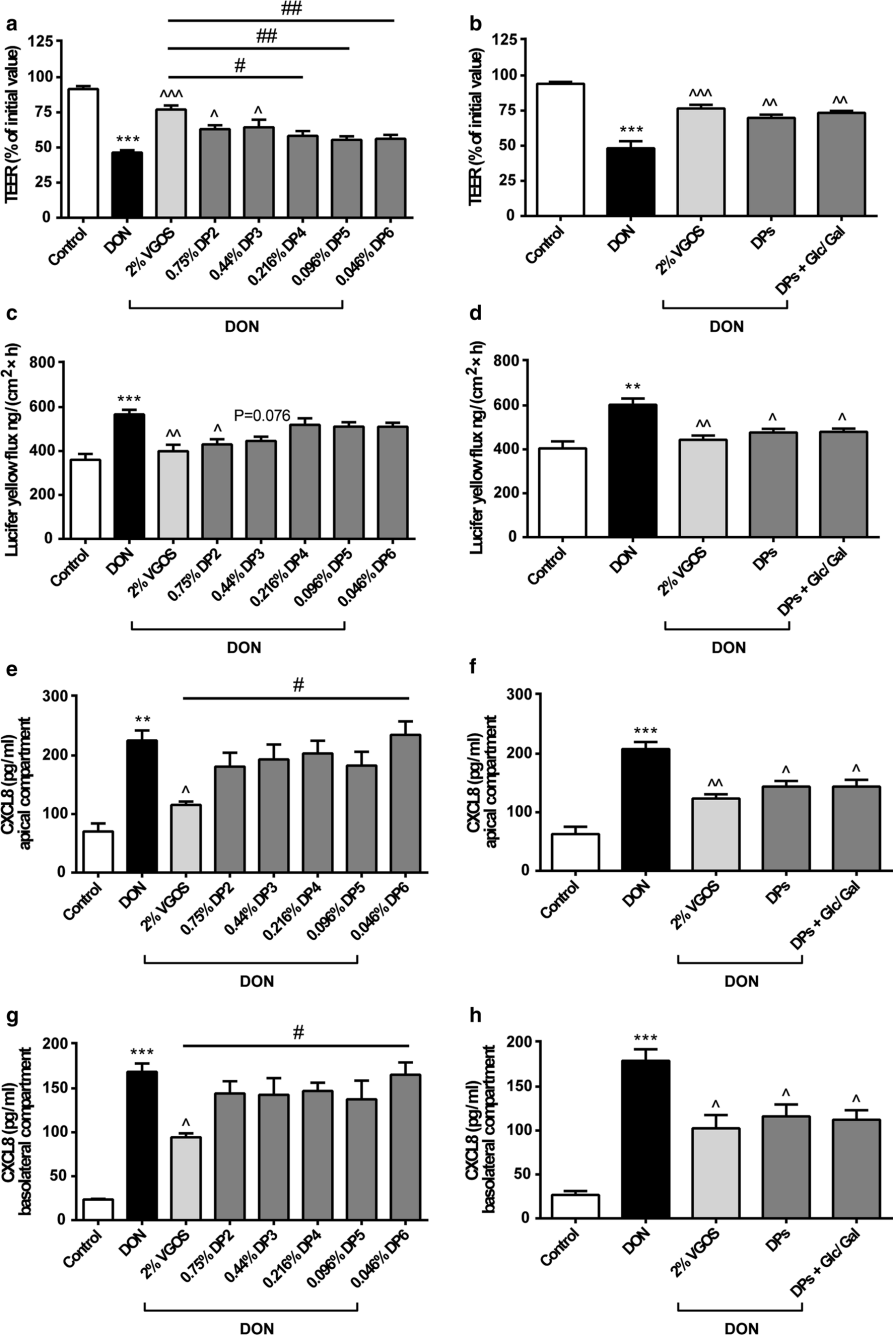
Combined DP fractions of VGOS mimicked VGOS in preventing DON-induced barrier disruption and CXCL8 release, whereas only individual DP2 and DP3 can prevent DON-induced barrier disruption. Caco-2 cells were pretreated apically and basolaterally with VGOS, individual DP fractions of GOS (ranging from DP2 to DP6) and combination of different DP fractions (DP2–DP6) with or without supplementation with glucose (Glc) and galactose (Gal) (24 h) prior to the addition of DON (4.2 μM) (apical and basolateral compartments) for 12 h. Subsequently, TEER (**a**, **b**), the transport of lucifer yellow (**c**, **d**) and CXCL8 release into the apical (**e**, **f**) and basolateral (**g**, **h**) compartment were measured. Results are expressed as a percentage of initial value (TEER), the amount of tracer transported [ng/(cm^2^ × h)] or pg/ml CXCL8 as mean ± SEM of three independent experiments, each performed in triplicate (**P < 0.01, ***P < 0.001: significantly different from the unstimulated cells; ^*P* < 0.05, ^^*P* < 0.01, ^^^*P* < 0.001: significantly different from the DON-stimulated cells; ^#^ *P* < 0.05, ^##^ *P* < 0.01: significantly different from each other)

**Fig. 6:**
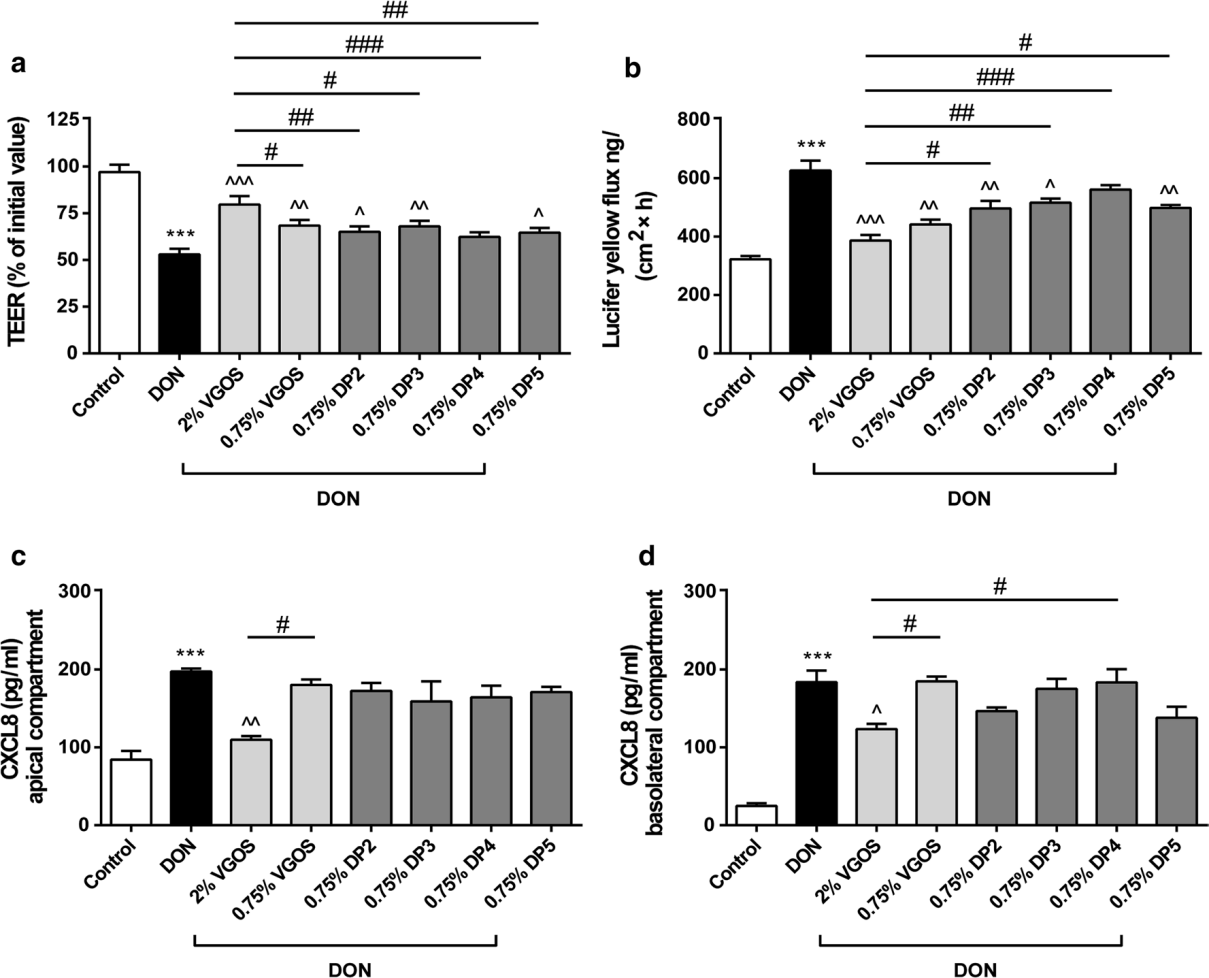
Different effects on the Caco-2 cell monolayer induced by individual DP fractions of VGOS with equal concentrations. Caco-2 cells were pretreated apically and basolaterally with VGOS (0.75 and 2 %) or individual DP fractions of VGOS (0.75 %, ranging from DP2 to DP5) (24 h) prior to the addition of DON (4.2 μM) (apical and basolateral compartments) for 12 h. Subsequently, TEER (**a**), the transport of lucifer yellow (**b**) and CXCL8 secretion into medium of apical (**c**) and basolateral (**d**) compartments were measured. Results are expressed as a percentage of initial value (TEER), the amount of tracer transported [ng/(cm^2^ × h)] or pg/ml CXCL8 as mean ± SEM and are representative of two independent experiments, each performed in triplicate (***P < 0.001: significantly different from the unstimulated cells; ^*P* < 0.05, ^^*P* < 0.01, ^^^*P* < 0.001: significantly different from the DON-stimulated cells; ^#^ *P* < 0.05, ^##^ *P* < 0.01, ^###^ *P* < 0.001: significantly different from each other)

**Fig. 7:**
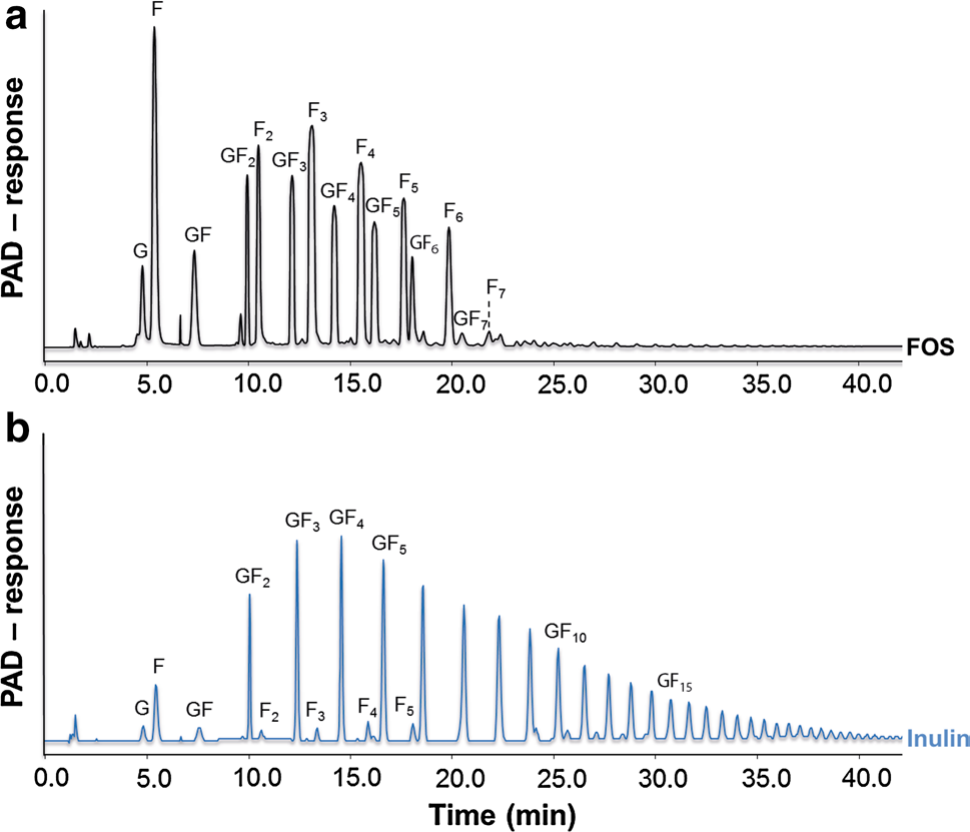
FOS and inulin characteristics. HPAEC-PAD elution patterns of FOS (**a**) and inulin (**b**). The *F*_*n*_ series represent oligomers consisting of fructose only, whereas the *GF*_*n*_ series represent fructose oligomers terminated with a terminal glucose molecule. The number of fructose units within an oligomer is indicated by _*n*_

**Fig. 8:**
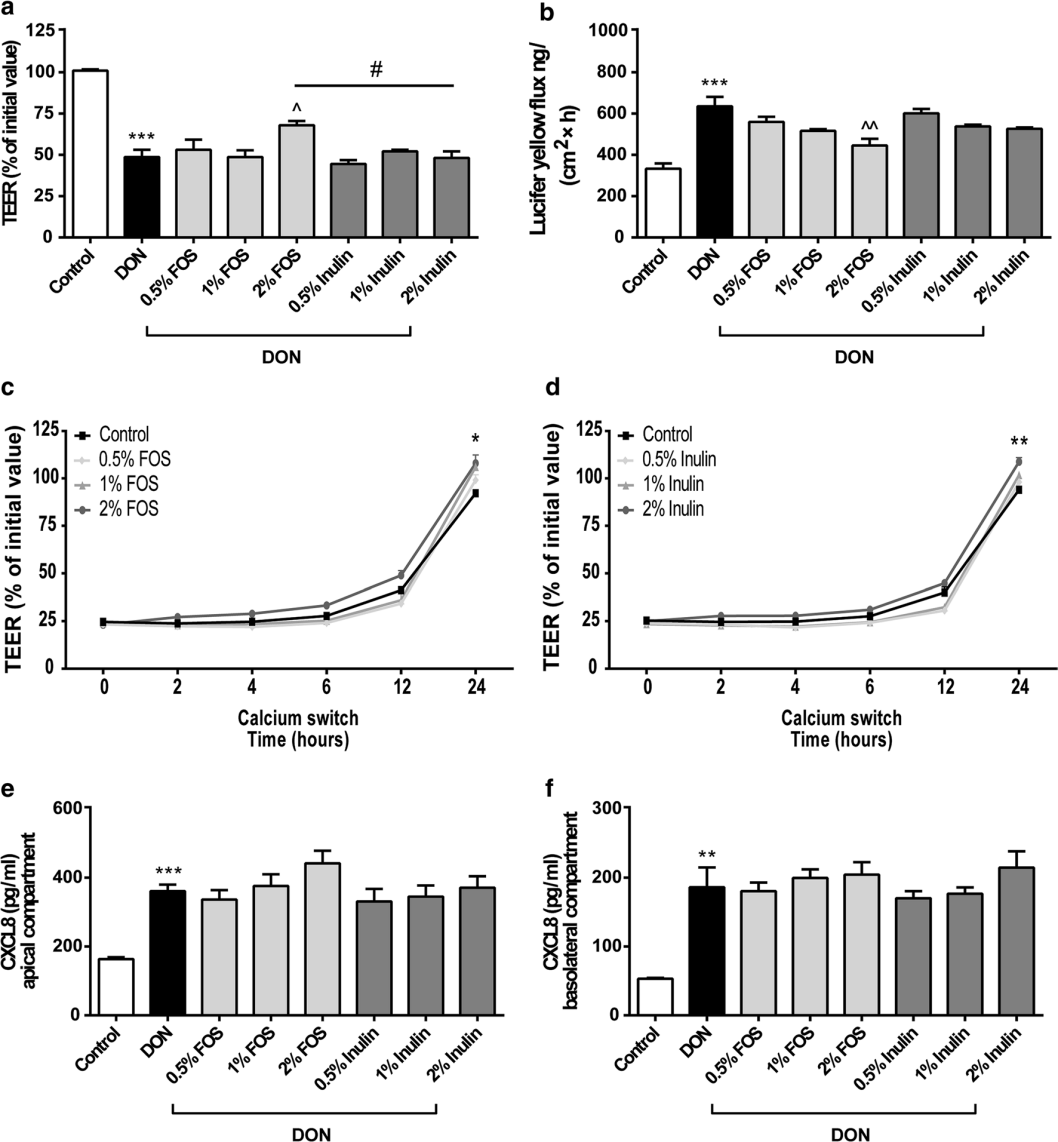
Different effects of FOS and inulin on the DON-induced barrier disruption, tight junction reassembly and CXCL8 release. Caco-2 cells were pretreated apically and basolaterally with increasing concentrations (0.5, 1 and 2 %) of FOS or inulin (24 h) prior to the addition of DON (4.2 μM) (apical and basolateral compartments) for another 24 h (**a**, **b**, **e**, **f**) or transient calcium deprivation with HBSS-EGTA to disrupt tight junction proteins (**c**, **d**). Subsequently, TEER values at the indicated time points (**a**, **c**, **d**), the transport of lucifer yellow (**b**) and CXCL8 secretion into medium of apical (**e**) and basolateral (**f**) compartments were measured. Results are expressed as a percentage of initial value (TEER), the amount of tracer transported [ng/(cm^2^ × h)] or pg/ml CXCL8 as mean ± SEM of three independent experiments, each performed in triplicate (*P < 0.05, **P < 0.01, ***P < 0.001: significantly different from the unstimulated cells; ^*P* < 0.05, ^^*P* < 0.01: significantly different from the DON-stimulated cells; ^#^ *P* < 0.05: significantly different from each other)

Galacto-oligosaccharides (Vivinal^®^ GOS syrup (VGOS, 59 % GOS on dry matter) and purified Vivinal^®^ GOS (PGOS, 97 % GOS on dry matter) without the monomeric sugars glucose, galactose and lactose were provided by FrieslandCampina Domo (Borculo, The Netherlands). Fructo-oligosaccharides (FOS, Orafti^®^P95) and inulin (Orafti^®^LGI) were obtained from Beneo Orafti (Tienen, Belgium). The detailed composition of the applied oligosaccharides is summarized in Online Resource 1 (related to Figs. 1, 2, 3, 4, 7, 8) and Online Resource 2 (related to Fig. 5, 6), and the oligosaccharide structures are schematically depicted in Online Resource 3. The concentrations used in this study are calculated based on the pure oligosaccharide fractions.

### Purification of GOS

Purified GOS with <3 % (w/w dry matter) monomers and lactose (purified from the lactose-based prebiotic Vivinal^®^ GOS syrup) were used. For the purification, Vivinal^®^ GOS syrup was enzymatically treated with a lactase to hydrolyze the lactose into glucose and galactose, after which the monosaccharides were removed by nanofiltration on laboratory scale [18]. By the purification process of Vivinal^®^ GOS syrup, next to lactose, also a part of the DP2 GOS is removed (Fig. 1).

### High-performance anion-exchange chromatography with pulsed amperometric detection (HPAEC-PAD)

Specific chain length profiles were characterized by HPAEC using a Dionex ICS 3000 system (Dionex, Sunnyvale, CA, USA), equipped with a Dionex CarboPac PA-1 column (2 × 250 mm) in combination with a CarboPac PA-1 guard column (2 × 50 mm) and a ISC5000 ED detector (Dionex) in the PAD mode as previously described [20, 21]. The individual peaks were characterized by comparing the obtained HPAEC-PAD profiles with SEC GOS-DP fractions and mass spectrometry.

### Size exclusion chromatography (SEC)

The oligosaccharides present in Vivinal^®^ GOS syrup were separated by SEC. Vivinal^®^ GOS syrup was fractionated using a XK50 column (length 60 cm, GE Healthcare, Pittsburgh, PA, USA) filled with Bio-Gel P-2 Gel Resin (Fine 45–90 μm, BioRad Laboratories, Hercules, CA, USA) connected to a AktaPrime Plus (GE Healthcare, Pittsburgh, PA, USA). The jacket of the column was connected to a water bath in order to maintain the temperature at 50 °C. Diluted Vivinal^®^ GOS syrup (approximately 30 % on DM) was injected onto the column (flowrate: 1.0 ml/min) using a 2-ml sample loop and eluted with demineralized water. Sampling was based on refractive index, and samples corresponding to the same DP were pooled and subsequently freeze-dried.

### The Caco-2 cell model

Human epithelial colorectal adenocarcinoma (Caco-2) cells obtained from American Type Tissue Collection (Code HTB-37) (Manassas, VA, USA, passages 90–102) were used according to established methods, also described by Akbari et al. [19]. In brief, cells were cultured in Dulbecco’s modified Eagle medium (DMEM) and seeded at a density of 0.3 × 10^5^ cells into 0.3 cm^2^ high pore density (0.4 μm) inserts with a polyethylene terephthalate membrane (BD Biosciences, Franklin Lakes, NJ, USA) placed in a 24-well plate. The Caco-2 cells were maintained in a humidified atmosphere of 95 % air and 5 % CO2 at 37 °C. After 17–19 days of culturing, a confluent monolayer was obtained with a mean TEER exceeding 400 Ω cm^2^ measured by a Millicell-Electrical Resistance System voltohmmeter (Millipore, Temecula, CA, USA).

Different concentrations (0.5, 1 and 2 %) of the oligosaccharides VGOS (v/v), PGOS (w/v), FOS (w/v) and inulin (w/v) and different DP fractions of VGOS [either in equimolar concentrations as present in VGOS or in a concentration of 0.75 % (w/v)] were prepared by dissolving in complete cell culture DMEM. Subsequently, the cells were preincubated with the different oligosaccharides or DP fractions of VGOS from apical side as well as basolateral side of the transwell inserts for 24 h before being challenged with DON in the presence of the different oligosaccharides or DP fractions of VGOS for another 24 or 12 h, respectively. Prior to the functional assays described below, the potential cytotoxicity of the different oligosaccharides or DP fractions of VGOS in Caco-2 cells had been evaluated by measuring lactate dehydrogenase (LDH) leakage (Promega Corp., Madison, WI, USA). These control experiments confirmed that none of the above oligosaccharides induced any cytotoxicity in Caco-2 cells at the concentrations used in the assays (data not shown).

### Transepithelial electrical resistance (TEER) measurement

The integrity of the Caco-2 cell monolayer grown on inserts was investigated by monitoring the TEER across the monolayer. A Millicell-ERS voltohmmeter connected to a pair of chopstick electrodes was used to measure the TEER values either 12 or 24 h after DON stimulation with or without pretreatment with the different oligosaccharides or DP fractions of VGOS for 24 h. Results are expressed as a percentage of the initial value.

### Paracellular tracer flux assay

Transport studies from the apical side to the basolateral side of a Caco-2 cell monolayer were performed using a membrane-impermeable molecule, lucifer yellow (LY, molecular mass: 0.457 kDa, Sigma, St. Luis, MO, USA). LY was added at a concentration of 16 μg/ml to the apical compartment (350 μl) in the transwell plate for 4 h, and the paracellular flux was determined by measuring the fluorescence intensity in the basolateral compartment with a spectrophotofluorimeter (FLUOstar Optima, BMG Labtech, Offenburg, Germany) set at excitation and emission wavelengths of 410 and 520 nm, respectively.

### Calcium switch assay

Caco-2 cells grown on inserts were pretreated with various concentrations (0.5, 1 and 2 %) of different oligosaccharides (VGOS, PGOS, FOS and inulin) on both sides of the transwell inserts for 24 h. Subsequently, Caco-2 cells were exposed transiently for 20 min to 2 mM ethylene glycol-bis(2-aminoethyl ether)*N*,*N*,*N*′,*N*′-tetraacetic acid (EGTA) (Sigma, St. Luis, MO, USA) in calcium- and magnesium-free Hanks’ balanced salt solution (HBSS) (Gibco, Invitrogen, Carlsbad, CA, USA) to disrupt tight junction proteins as previously described [8]. At the end of the incubation period, the cells were rinsed and allowed to recover either in complete cell culture DMEM (containing 2 mM CaCl_2_) or in DMEM supplemented with the addition of various concentrations (0.5, 1 and 2 %) of the different oligosaccharides. During this recovery period, reassembly of tight junctions and restoration of barrier function were determined by measuring the TEER at various time points (2, 4, 6, 12 and 24 h). The results are expressed as a percentage of initial value.

### CXCL8 secretion

The inflammatory marker, CXCL8, was quantified in the medium of the apical side and the basolateral side of the Caco-2 transwell inserts in response to the treatments. CXCL8 concentrations were measured by using the human IL-8 ELISA (BD Biosciences, Pharmingen, San Diego, CA, USA) according to manufacturer’s instructions.

### Statistical analysis

Experimental results are expressed as mean ± SEM. Analyses were performed by using GraphPad Prism (version 6.05) (GraphPad, La Jolla, CA, USA). Differences between groups were statistically determined by using one-way ANOVA, with Bonferroni’s post hoc test. Results were considered statistically significant when *P* < 0.05.

## Results

### VGOS and PGOS characteristics

The HPAEC-PAD chromatograms of VGOS and PGOS (Fig. 1) clearly demonstrated that both GOS samples are complex mixtures of oligosaccharides with a DP of mainly 2–6 and various isomers per DP (Online Resource 3). PGOS, derived from VGOS, was predominantly lacking the monosaccharides and lactose, although also some other components of GOS-DP2 were (partly) removed.

### Different effects of VGOS and PGOS on the DON-induced impairment of the Caco-2 cell monolayer integrity

Pretreatment with VGOS prevented the DON-induced decrease in TEER values in a concentration-dependent manner as depicted in Fig. 2a, while only the highest concentration of PGOS significantly attenuated the DON-induced TEER decrease; however, this effect was significantly lower compared with 2 % VGOS (Fig. 2a). In line with the effects on the observed TEER values, the DON-induced increase in tracer transport (LY) was significantly decreased by 1 % VGOS, 2 % VGOS and 2 % PGOS (Fig. 2b).

### VGOS time-dependently accelerates tight junction reassembly after calcium deprivation in Caco-2 cells

It was demonstrated that 2 % VGOS caused a time-dependent acceleration in tight junction reassembly over a period of 24 h (Fig. 3a), and the first significant effect on TEER restoration was already observed 4 h after calcium recovery. In addition, 1 % VGOS also showed a significant improvement in TEER values after 24-h recovery. Caco-2 cells incubated with 2 % PGOS showed only improved restoration in TEER after 24-h recovery, whereas the other tested PGOS concentrations (0.5 and 1 %) did not accelerate the tight junction reassembly (Fig. 3b).

### VGOS and not PGOS is able to suppress the DON-induced increase in CXCL8 release by Caco-2 cells

The DON-induced increased levels of secreted CXCL8 could be only prevented by preincubation with 1 and 2 % VGOS as observed by a concentration-dependent decrease in CXCL8 release on both sides (Fig. 4a, b). PGOS exposure to Caco-2 cells did not affect CXCL8 secretion into neither the apical nor the basolateral compartment of the transwell system (Fig. 4a, b).

### Supplementation of glucose, galactose and lactose to PGOS does not mimic the effect of VGOS against DON-induced barrier disruption and CXCL8 release

Supplementation of glucose, galactose and lactose (in equimolar concentrations as present in 2 % VGOS, Online Resource 2) to 2 % PGOS did not improve the effect of PGOS on the DON-induced gut barrier impairment as observed by TEER and paracellular flux of LY (Online Resource 4). In addition, this supplementation to PGOS did not mimic the protective effect of VGOS against the DON-induced increase in CXCL8 release (Online Resource 4).

### Combined DP fractions of VGOS mimic VGOS in preventing DON-induced barrier disruption and CXCL8 release, whereas only individual DP2 and DP3 prevent the DON-induced barrier disruption

Pretreatment with DP2 or DP3 (in equimolar concentrations as present in VGOS) prevented the DON-induced decrease in TEER values (Fig. 5a). The DON-induced increase in tracer transport was significantly decreased by DP2, whereas the effect of DP3 was not significantly different (*P* = 0.076) (Fig. 5c). None of the individual DP4, DP5 or DP6 induced a preventive effect on the DON-induced intestinal epithelial barrier impairment as observed by TEER (Fig. 5a) and paracellular flux of LY (Fig. 5c), and none of the individual DP fractions (ranging from DP2 to DP6) was able to prevent the DON-induced increase in secreted CXCL8 (Fig. 5e, g). The combination of different DP fractions of GOS (ranging from DP2 to DP6 in equimolar concentrations as present in VGOS) with or without glucose and galactose supplementation did show a protective effect against DON-induced gut barrier impairment and CXCL8 release comparable to the effect of VGOS (Fig. 5b, d, f, h). In addition, the effects of individual DP fractions and the combined DP fractions were also determined in non-treated Caco-2 cells. These results indicated that neither individual DP fractions nor the combination of different DP fractions with or without glucose and galactose supplementation affects gut barrier function and CXCL8 release (Online Resource 5).

### Different effects on the Caco-2 cell monolayer induced by individual DP fractions of VGOS with equal concentrations

Next to investigating the effect of DPs in equimolar concentrations as present in VGOS, we studied the effects of the individual DP fractions in equal concentrations of 0.75 % to clarify concentration-dependent effects of DP fractions. Pretreatment with 0.75 % of individual DP2, DP3 or DP5 significantly prevented the DON-induced decrease in TEER values and DON-induced increase in tracer transport (Fig. 6a, b), and this effect was similar to that of 0.75 % VGOS (Fig. 6a, b). DP4 (0.75 %) did not significantly affect the DON-induced gut barrier impairment as measured by TEER (Fig. 6a) and paracellular flux of LY (Fig. 6b). In addition, neither 0.75 % VGOS nor the individual DP fractions (0.75 %, ranging from DP2 to DP5) attenuated the DON-induced increase in CXCL8 release (Fig. 6c, d).

### FOS and inulin characteristics

Figure 7 shows the HPAEC-PAD chromatograms of FOS (a) and inulin (b) and demonstrates that FOS clearly contain oligosaccharides up to DP7–8, built up by fructose residues (*F*_*n*_ series) and by fructose residues with a terminally linked glucose moiety (*GF*_*n*_ series). Inulin mainly consists of fructose chains terminated with a glucose molecule representing a wide range of DPs (Online Resource 3).

### Different effects of FOS and inulin on the DON-induced barrier disruption, tight junction reassembly and CXCL8 release

Furthermore, the microbiota-independent effects of GOS were compared to oligosaccharides with a different structure and DP, including inulin and FOS. The highest concentration of FOS (2 %) significantly modulated the DON-induced epithelial barrier disruption as measured by TEER values and paracellular flux of LY, whereas none of the tested inulin concentrations induced a preventive effect on the barrier integrity of the Caco-2 monolayer (Fig. 8a, b). Caco-2 cells incubated with 2 % FOS or 2 % inulin showed both improved restoration in TEER 24 h after calcium recovery, whereas the other tested concentrations (0.5 and 1 %) did not accelerate the tight junction reassembly after calcium deprivation (Fig. 8c, d). DON-induced increase in CXCL8 secretion was affected by neither FOS nor inulin (Fig. 8e, f).

## Discussion

Non-digestible oligosaccharides, including GOS and mixtures of GOS/lcFOS, are commonly used in infant formula as an alternative for human milk oligosaccharides, which are not commercially available [12, 22]. Gut health-promoting effects of GOS and GOS/lcFOS are not limited to the modulation of the intestinal microbiota, since also direct interaction between intestinal epithelial cells (IEC) and these oligosaccharides has recently been described [8–11]. The prebiotic activity of GOS and FOS is believed to be determined by their unique structure and specific DP composition [12, 13, 17, 18]. However, the effect of structure and DP composition of GOS or FOS on direct interaction with IEC has never been studied. Hence, in the current study the direct effects of Vivinal^®^ GOS syrup, purified Vivinal^®^ GOS and their DP fractions were compared in a DON-stimulated Caco-2 cell model for intestinal barrier dysfunction as previously described [19]. We aimed to understand which DP fractions of VGOS are responsible for the observed protective effects. Furthermore, the effects of oligosaccharides with a different structure and DP than GOS, such as inulin and FOS, were examined in this Caco-2 cell model.

The mycotoxin DON was used as a model compound to impair the intestinal integrity. DON is a 12,13-epoxytrichothecene and is known to directly impair tight junction integrity and to induce an inflammatory response [19, 23, 24]. Our group recently found that GOS stimulate the tight junction reassembly and in turn mitigate the deleterious effects of DON on the intestinal barrier of Caco-2 cells and this effect is not related to a direct interaction between DON and GOS [8].

GOS are derived from lactose and consist of a chain of galactose units with a terminal glucose monomer, different glycosidic linkages (e.g., *β*(1–4) and *β*(1–6)), and the DP varies between 2 and 8 [14, 25]. GOS structures have the lactose building block in common with HMOs, although the latter is more complex, since HMO oligomers may also have galactose and N-acetylglucosamine (GlcNAc) in their backbone, which are further substituted with fructose and neuraminic acid [2, 26]. Holscher et al. [27] reported different roles for specific HMOs in the differentiation and barrier function of Caco-2 cells.

In the current study, VGOS was most effective and purified GOS (PGOS) was significantly less effective with respect to the improvement of the impairment of the Caco-2 cell monolayer integrity as well as the modulation of immune responses, including CXCL8 release. The monosaccharides (glucose and galactose) and disaccharide (lactose) present in Vivinal^®^ GOS syrup were not responsible for this superior effect of VGOS over PGOS, since combined supplementation of glucose, galactose and lactose to PGOS did not mimic the effect of VGOS against the DON-induced effects, as shown by the current experiments. We previously confirmed that similar concentrations of these saccharides present in the 2 % GOS solution did not affect the DON-induced impairment of Caco-2 monolayer integrity [8]. Comparing the HPAEC elution patterns (Fig. 1), another difference between VGOS and PGOS is the amount of DP2, which is partly lost during the purification process. It could therefore be hypothesized that DP2 is driving the higher potency of VGOS. In our experiments, we demonstrated that isolated DP2 and DP3 fractions (in equimolar concentrations as present in VGOS) significantly prevented the DON-induced barrier disruption, whereas no effect of the larger DPs (equal or above DP4) was observed. The prominent effect of DP2 and DP3 seems to be related to their high prevalence in the VGOS mixture. When we tested equal concentrations (0.75 %) of all available DPs, only DP4 was not effective in preventing the Caco-2 monolayer disruption by DON.

Related to our results, the DP concentration and to a lesser extent the DP length may be crucial for the observed protective effects of galacto-oligosaccharides. Previous studies had already indicated that the effect of individual oligosaccharides on the intestinal microbiota can be related to their DP composition. Ladirat et al. [18] described that the addition of GOS or a DP3 fraction to a human fecal inoculum resulted in the highest *Bifidobacterium* spp. increase, whereas the DP4 and DP5 fractions displayed the lowest increase. In line with our results, they did not find significant differences between the DP GOS fractions and VGOS at similar concentrations. In a study with maltose-based oligosaccharides, DP3 oligomers showed the highest selectivity toward bifidobacteria, and oligosaccharides above DP7 did not promote the growth of beneficial bacteria, including bifidobacteria [13]. In contrast, the presence of the larger DP fractions within GOS was effective in restoring the microbiota following an antibiotic-induced dysbacteriosis [18], whereas Sinclair et al. [28] showed that in particular higher DP GOS fractions were capable of inhibiting the in vitro binding of *Vibrio cholerae* toxin to its GM1 receptor. We therefore speculate that DP length of oligosaccharides is related to different physiological effects.

In contrast to galacto-oligosaccharides, inulin (or lcFOS) consists of a mixture of fructose residues linked by *β*(2–1) fructosyl-fructose glycosidic bonds with a glucose monomer at the end of almost each fructose chain within a DP range of 2–60. FOS have been produced from inulin by partial enzymatic hydrolysis and differ from inulin to its degree of polymerization (DP2-8), and fructose oligomers occur with or without the presence of a terminal glucose moiety (Fig. 7) [16, 20]. Schematic structures of GOS, FOS and inulin are depicted in Online Resource 3. Only 2 % FOS showed moderate, but significant, protective effects against intestinal barrier dysfunction as observed by TEER recovery and decreased LY paracellular flux, whereas none of the mentioned parameters were affected by inulin. Since the DP of both GOS and FOS varies from 2 to 8, we can speculate that these smaller DP fractions (DP2–8) are important for inducing the protective effects on barrier integrity compared with higher DP fractions DP9-60. Also in a different experimental setting, Shoaf et al. [29] described that GOS were most effective in preventing bacterial colonization and pathogen invasion by their anti-adhesive capacity compared with the other oligosaccharides, including FOS, inulin, lactulose and raffinose.

With regard to the potential anti-inflammatory properties of oligosaccharides, our results demonstrate that only VGOS was able to prevent the DON-induced CXCL8 release, whereas PGOS, the separate GOS-DP fractions, FOS and inulin did not prevent CXCL8 secretion. The anti-inflammatory activity of VGOS was also observed by Vendrig et al. [30] since peripheral blood mononuclear cells (PBMCs) derived from VGOS-treated foals were less responsive to a lipopolysaccharide challenge and did show lower IFN-γ and IL-6 mRNA expression levels. Differences in chain length between different FOS-products could be responsible for differential effects in skewing the cytokine balance, since Vogt et al. [20] found a positive correlation between chain length of FOS and IL-10/IL-12 ratios in human PBMCs. Different in vitro and ex vivo studies displayed that short-chain FOS and long-chain FOS are able to induce a more anti-inflammatory or pro-inflammatory condition, respectively [20, 31, 32]. In addition, higher concentrations of inulin or short-chain FOS (10 %) did significantly decrease the CXCL8 gene expression induced by *Citrobacter rodentium* [33], and in unstimulated Caco-2 cells, FOS reduced the CXCL8, IL-12 and TNF-α gene expression via activation of peptidoglycan recognition protein 3 (PGlyRP3) and peroxisome proliferator-activated receptor γ (PPARγ) [11]. In the presence of a GOS/lcFOS mixture, a potential role for Toll-like receptor (TLR)-9 and galectin-9 in modulating cytokine production has been demonstrated [34, 35]. In contrast, Ortega-González et al. [36] hypothesized that GOS and FOS are TLR4 ligands in intestinal epithelial cells, which may be a relevant mechanism for the immunomodulatory effects of prebiotics. However, this latter mechanism cannot explain our results in the Caco-2 cell model, since these cells do not express TLR4 [37]. The direct effect of VGOS on cytokine expression and release is possibly mediated by other pattern recognition receptor(s), and this signaling pathway seems to be galacto-oligosaccharide-specific.

In summary, this study for the first time compared direct, microbiota-independent effects, of defined oligosaccharides, including GOS (Vivinal^®^ GOS syrup (VGOS) and purified Vivinal^®^ GOS (PGOS)), and their related DPs, as well the plant-derived oligosaccharides FOS and inulin on intestinal epithelial cells. It can be concluded that the tested oligosaccharides have different capacities to regulate the DON-disrupted epithelial monolayer and the related immune response. VGOS showed the most significant protective effect on all tested parameters in a concentration-dependent manner. Overall, differences in oligosaccharide structure and size result in significant changes in the direct, microbiota-independent effects.

## Electronic supplementary material

Below is the link to the electronic supplementary material.

Supplementary material 1 (PDF 85 kb)

Supplementary material 2 (PDF 85 kb)

Supplementary material 3 (PDF 45 kb)

Supplementary material 4 (PDF 256 kb)

Supplementary material 5 (PDF 229 kb)

## Abbreviations

DMEM: Dulbecco’s modified eagle medium
DON: Deoxynivalenol
DP: Degree of polymerization
*F*_*n*_: Fructose oligomers
FOS: Fructo-oligosaccharides
Gal: Galactose
*GF*_*n*_: Fructose oligomers terminated with a glucose molecule
Glc: Glucose
GlcNAc: N-acetylglucosamine
GOS: Galacto-oligosaccharides
HMOs: Human milk oligosaccharides
HPAEC-PAD: High-performance anion-exchange chromatography with pulsed amperometric detection
IEC: Intestinal epithelial cells
IL8/CXCL8: Interleukin-8
Lac: Lactose
lcFOS: Long-chain FOS or inulin
LY: Lucifer yellow
PBMCs: Peripheral blood mononuclear cells
PGOS: Purified Vivinal^®^ GOS
TEER: Transepithelial electrical resistance
VGOS: Vivinal^®^ GOS syrup
Wt %: Weight percent
