# Supplementary material for: Characterizing microbiota-independent effects of oligosaccharides on intestinal epithelial cells: insight into the role of structure and size: Structure–activity relationships of non-digestible oligosaccharides
Source: Eur J Nutr. 2016 Jun 13;56(5):1919–30. doi: 10.1007/s00394-016-1234-9 (PMC5534205; doi:10.1007/s00394-016-1234-9)
Supplement: Supplementary file 1 — Supplementary material 1 (PDF 85 kb) [file 394_2016_1234_MOESM1_ESM.pdf]

Article title: Characterizing microbiota-independent effects of oligosaccharides on intestinal epithelial cells: insight into the role of structure and size

Journal name: European Journal of Nutrition

Authors: Peyman Akbari, Johanna Fink-Gremmels, Rianne H.A.M. Willems, Elisabetta Difilippo, Henk A. Schols, Margriet H.C. Schoterman, Johan Garssen, Saskia Braber

Corresponding author:
Saskia Braber
Utrecht University, Yalelaan 104, 3584 CM Utrecht, The Netherlands
Phone: +31 30 2531078, Fax: +31 30 2535700
E-mail: S.braber@uu.nl

Online Resource 1
Characteristics of the applied oligosaccharides

Products	Dry matter	Oligosaccharides (wt % on DM)	Mono-and Di-saccharides (w/w)	DP
**VGOS**	75%	59	glucose, galactose, lactose: 41%	2-8
**PGOS**	>98%	97	glucose, galactose, lactose: 3%	2-8
**FOS**	>98%	93.2	glucose, fructose, sucrose: 6.8%	2-8
**Inulin**	>98%	96	glucose, fructose, sucrose: 4%	2-60
