# Supplementary material for: Characterizing microbiota-independent effects of oligosaccharides on intestinal epithelial cells: insight into the role of structure and size: Structure–activity relationships of non-digestible oligosaccharides
Source: Eur J Nutr. 2016 Jun 13;56(5):1919–30. doi: 10.1007/s00394-016-1234-9 (PMC5534205; doi:10.1007/s00394-016-1234-9)
Supplement: Supplementary file 2 — Supplementary material 2 (PDF 85 kb) [file 394_2016_1234_MOESM2_ESM.pdf]

Article title: Characterizing microbiota-independent effects of oligosaccharides on intestinal epithelial cells: insight into the role of structure and size

Journal name: European Journal of Nutrition

Authors: Peyman Akbari, Johanna Fink-Gremmels, Rianne H.A.M. Willems, Elisabetta Difilippo, Henk A. Schols, Margriet H.C. Schoterman, Johan Garssen, Saskia Braber

Corresponding author:
Saskia Braber
Utrecht University, Yalelaan 104, 3584 CM Utrecht, The Netherlands
Phone: +31 30 2531078, Fax: +31 30 2535700
E-mail: S.braber@uu.nl

Online Resource 2
Percentages of DP composition in VGOS

Compound	wt % (on DM)
**Galacto-oligosaccharides**	61.9
**DP2 (including lactose)**	37.8
**DP3**	22.0
**DP4**	10.8
**DP5**	4.8
**DP6 and higher**	2.3
**Lactose in DP2**	15.8
**Glucose**	20.7
**Galactose**	1.1
**Total**	99.5%
