# Supplementary material for: Characterizing microbiota-independent effects of oligosaccharides on intestinal epithelial cells: insight into the role of structure and size: Structure–activity relationships of non-digestible oligosaccharides
Source: Eur J Nutr. 2016 Jun 13;56(5):1919–30. doi: 10.1007/s00394-016-1234-9 (PMC5534205; doi:10.1007/s00394-016-1234-9)
Supplement: Supplementary file 3 — Supplementary material 3 (PDF 45 kb) [file 394_2016_1234_MOESM3_ESM.pdf]

Article title: Characterizing microbiota-independent effects of oligosaccharides on intestinal epithelial cells: insight into the role of structure and size

Journal name: European Journal of Nutrition

Authors: Peyman Akbari, Johanna Fink-Gremmels, Rianne H.A.M. Willems, Elisabetta Difilippo, Henk A. Schols, Margriet H.C. Schoterman, Johan Garssen, Saskia Braber

Corresponding author:
Saskia Braber
Utrecht University, Yalelaan 104, 3584 CM Utrecht, The Netherlands
Phone: +31 30 2531078, Fax: +31 30 2535700
E-mail: S.braber@uu.nl

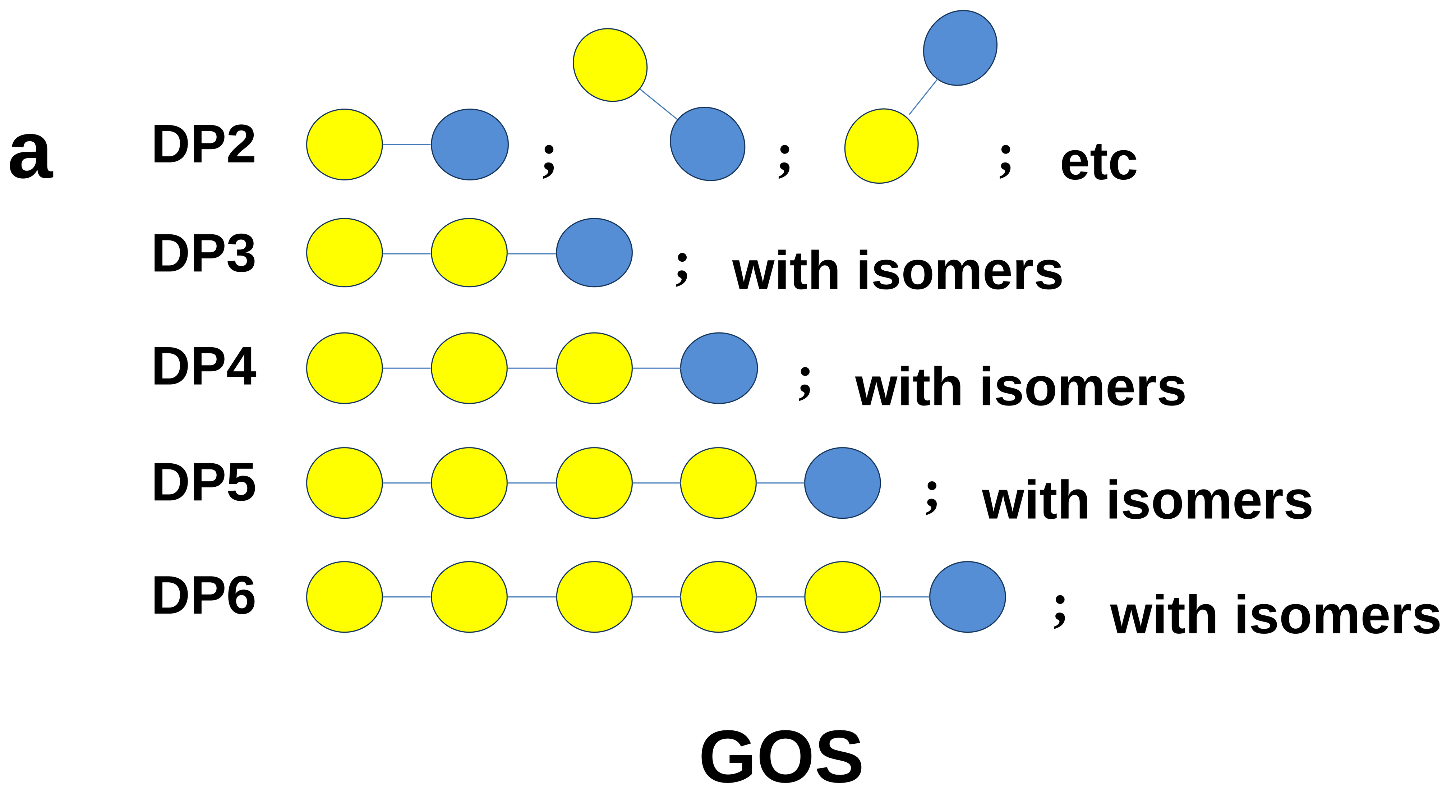

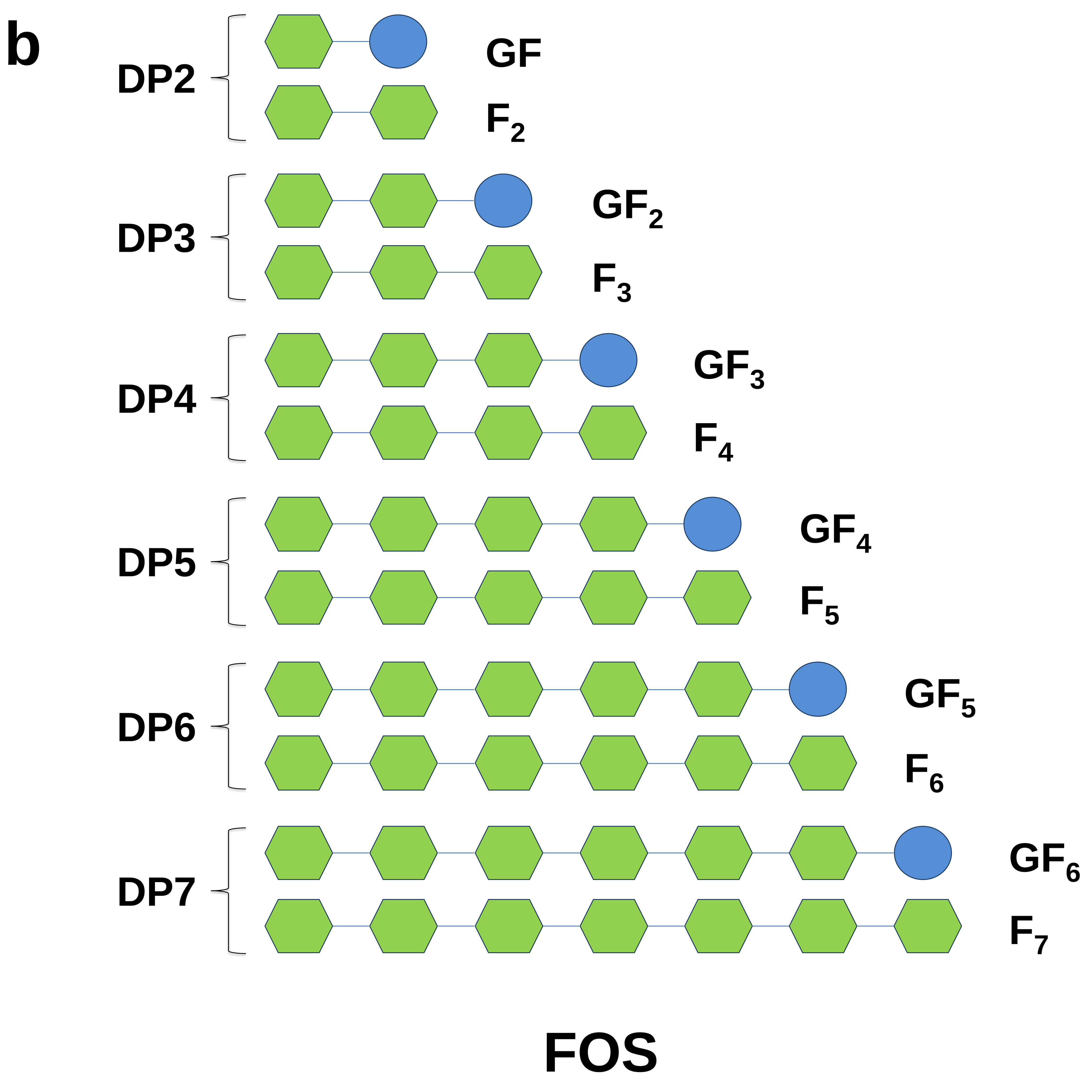

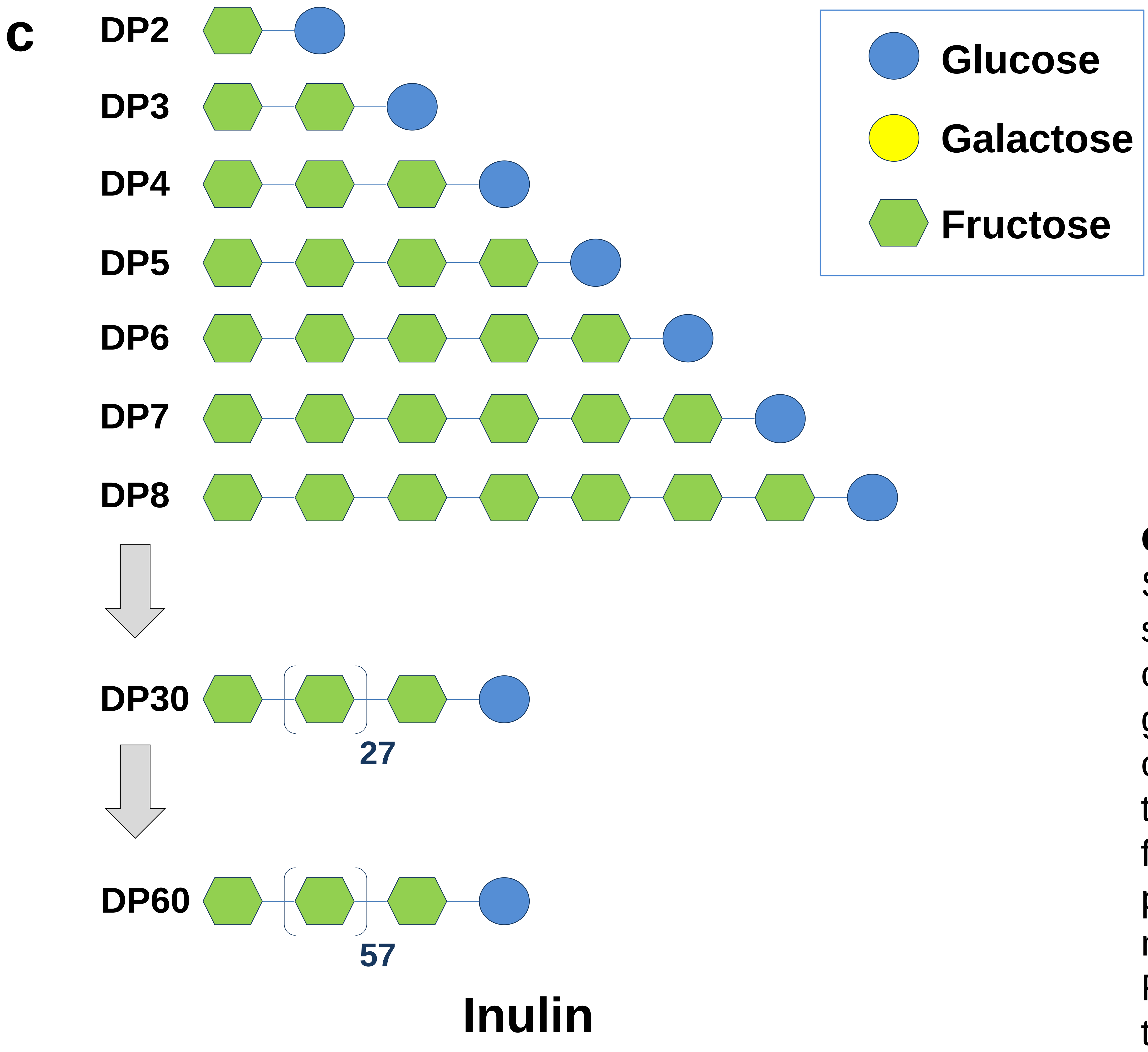

Online Resource 3

Schematic representation of the main oligosaccharide structures present in GOS, FOS and Inulin. GOS are comprising of a chain of galactose units with a terminal glucose monomer (**a**). FOS contain both F_n + GF_n series of oligomers (**b**), while Inulin mainly consists of the GF_n type fructans (**c**). The total number of galactose or fructose with terminal glucose units (degree of polymerization, DP) of GOS, FOS and Inulin ranges mainly between 2-6, 2-7 and 2-60, respectively.

F_n = number of fructose subunits, GF_n = fructose chain terminated with a glucose molecule.
