# Supplementary material for: Characterizing microbiota-independent effects of oligosaccharides on intestinal epithelial cells: insight into the role of structure and size: Structure–activity relationships of non-digestible oligosaccharides
Source: Eur J Nutr. 2016 Jun 13;56(5):1919–30. doi: 10.1007/s00394-016-1234-9 (PMC5534205; doi:10.1007/s00394-016-1234-9)
Supplement: Supplementary file 4 — Supplementary material 4 (PDF 256 kb) [file 394_2016_1234_MOESM4_ESM.pdf]

Article title: Characterizing microbiota-independent effects of oligosaccharides on intestinal epithelial cells: insight into the role of structure and size

Journal name: European Journal of Nutrition

Authors: Peyman Akbari, Johanna Fink-Gremmels, Rianne H.A.M. Willems, Elisabetta Difilippo, Henk A. Schols, Margriet H.C. Schoterman, Johan Garssen, Saskia Braber

Corresponding author:
Saskia Braber
Utrecht University, Yalelaan 104, 3584 CM Utrecht, The Netherlands
Phone: +31 30 2531078, Fax: +31 30 2535700
E-mail: S.braber@uu.nl

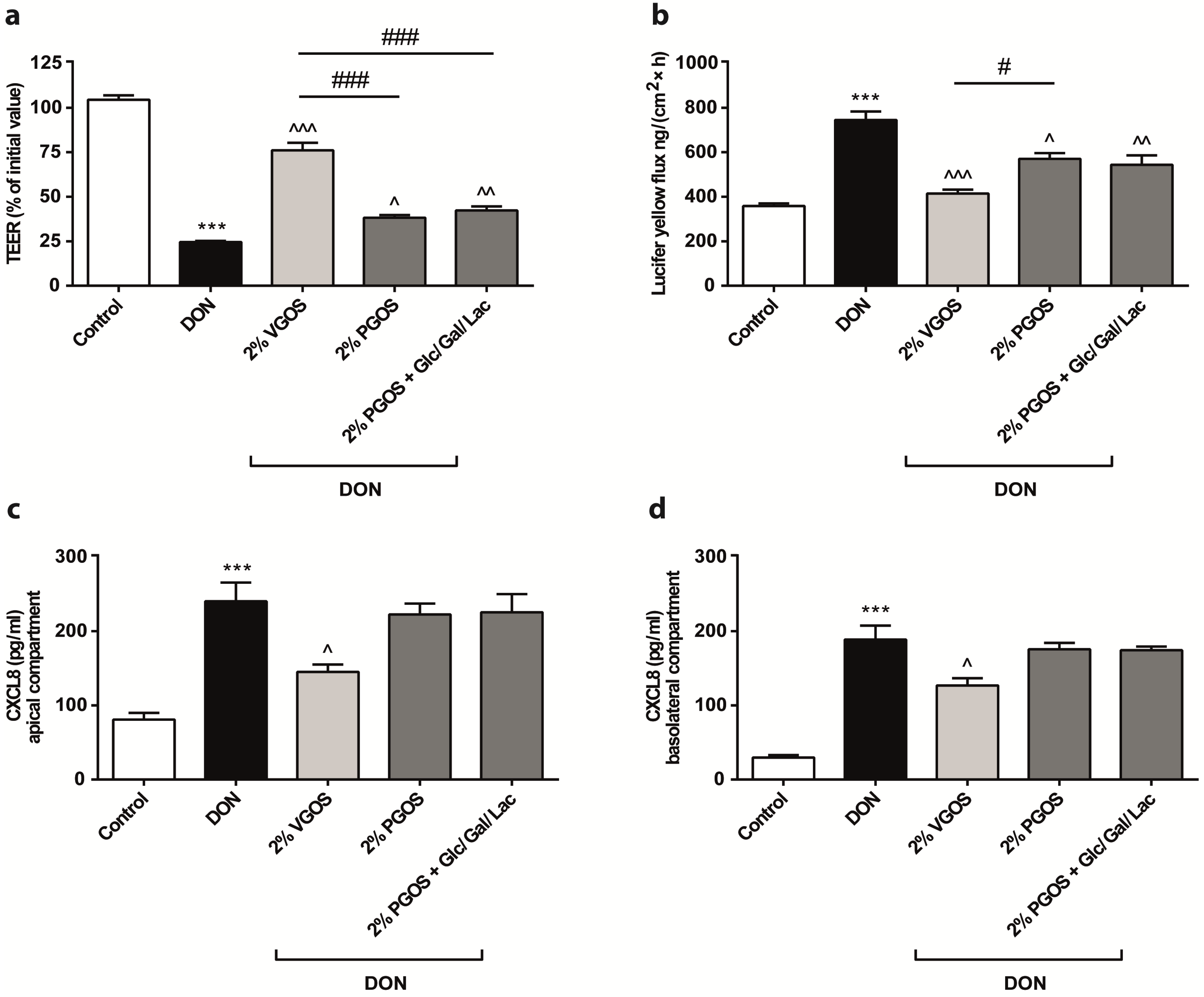

Online Resource 4

Supplementation of glucose, galactose and lactose to PGOS did not mimic the effect of VGOS against DON-induced barrier disruption and CXCL8 release. Caco-2 cells were pretreated apically and basolaterally with VGOS and PGOS with or without supplementation with glucose (Glc), galactose (Gal) and lactose (Lac) (24 h) prior to the addition of DON (4.2 µM) (apical and basolateral compartments) for 24 h. Subsequently, TEER (**a**), the transport of lucifer yellow (**b**) and CXCL8 release into the apical (**c**) and basolateral (**d**) compartment were measured. Results are expressed as a percentage of initial value (TEER), the amount of tracer transported [ng/(cm^2 × h)] or pg/ml CXCL8 as means ± SEM of three independent experiments, each performed in triplicate (***P < 0.001; significantly different from the unstimulated cells. ^P < 0.05, ^^P < 0.01, ^^^P < 0.001; significantly different from the DON-stimulated cells. #P < 0.05, ###P < 0.001; significantly different from each other).
