# Supplementary material for: Characterizing microbiota-independent effects of oligosaccharides on intestinal epithelial cells: insight into the role of structure and size: Structure–activity relationships of non-digestible oligosaccharides
Source: Eur J Nutr. 2016 Jun 13;56(5):1919–30. doi: 10.1007/s00394-016-1234-9 (PMC5534205; doi:10.1007/s00394-016-1234-9)
Supplement: Supplementary file 5 — Supplementary material 5 (PDF 229 kb) [file 394_2016_1234_MOESM5_ESM.pdf]

Article title: Characterizing microbiota-independent effects of oligosaccharides on intestinal epithelial cells: insight into the role of structure and size

Journal name: European Journal of Nutrition

Authors: Peyman Akbari, Johanna Fink-Gremmels, Rianne H.A.M. Willems, Elisabetta Difilippo, Henk A. Schols, Margriet H.C. Schoterman, Johan Garssen, Saskia Braber

Corresponding author:
Saskia Braber
Utrecht University, Yalelaan 104, 3584 CM Utrecht, The Netherlands
Phone: +31 30 2531078, Fax: +31 30 2535700
E-mail: S.braber@uu.nl

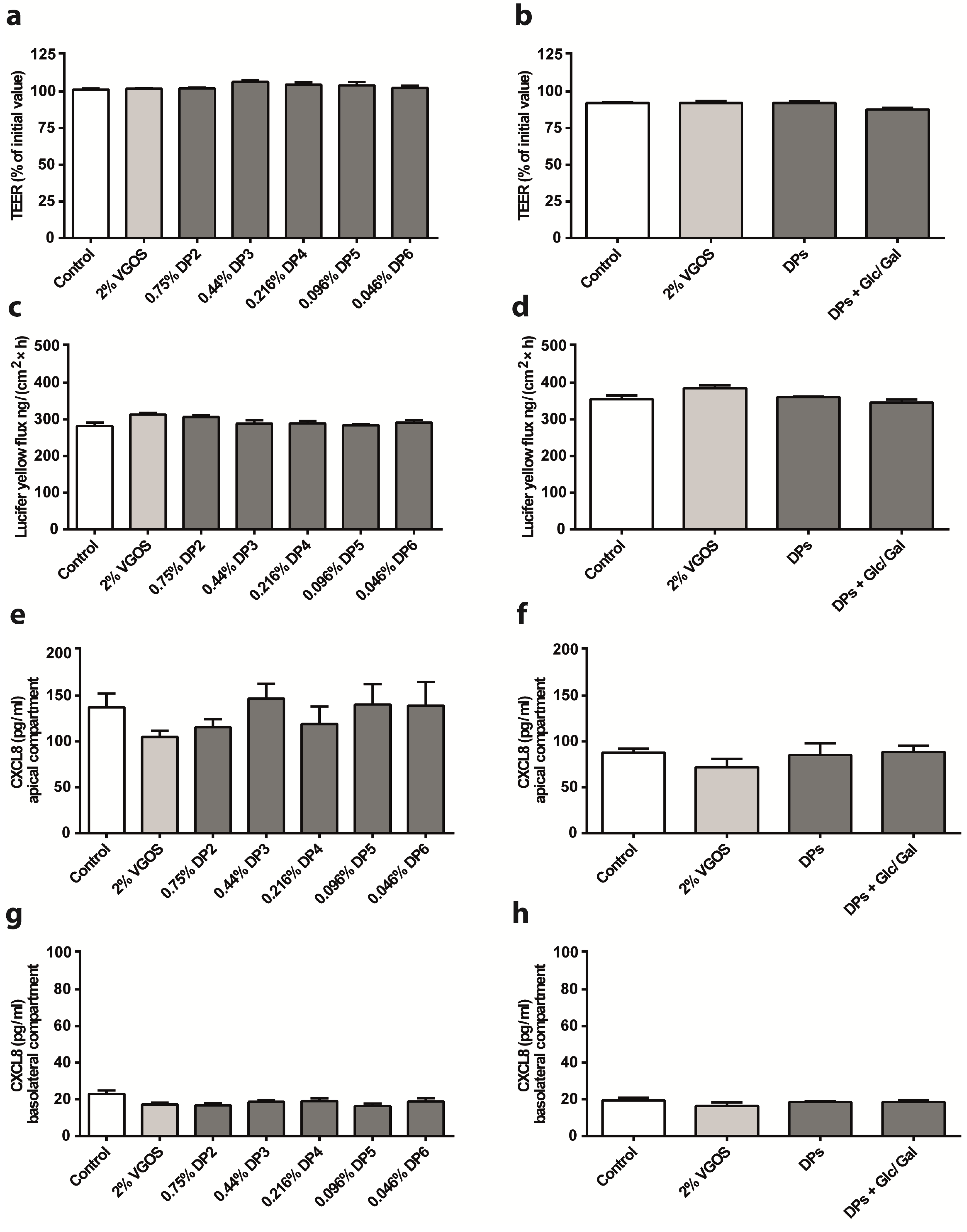

Online Resource 5

The effects of individual and combined DP fractions of VGOS on the gut barrier function and CXCL8 release by Caco-2 cells. Caco-2 cells were incubated apically and basolaterally with VGOS, individual DP fractions of GOS (ranging from DP2 to DP6) and combination of different DP fractions (DP2 to DP6) with or without supplementation with glucose (Glc) and galactose (Gal) (36 h). Subsequently, TEER (**a**, **b**), the transport of lucifer yellow (**c**, **d**) and CXCL8 release into the apical (**e**, **f**) and basolateral (**g**, **h**) compartment were measured. Results are expressed as a percentage of initial value (TEER), the amount of tracer transported [ng/(cm^2 × h)] or pg/ml (ELISA) as means ± SEM, n = 1 (performed in triplicate).
